# Determinants of bank’s efficiency in an emerging economy: A data envelopment analysis approach

**DOI:** 10.1371/journal.pone.0281663

**Published:** 2023-03-14

**Authors:** Saif Ullah, Abdul Majeed, József Popp

**Affiliations:** 1 Department of Management, Technology and Information Sciences, Faculty of Engineering, Sciences, Technology and Management at Ziauddin University, Karachi, Pakistan; 2 Business School, Huanggang Normal University, Huanggang, Hubei, China; 3 John von Neumann University, Hungarian National Bank–Research Center, Kecskemét, Hungary; 4 College of Business and Economics, University of Johannesburg, Johannesburg, South Africa; Rzeszow University of Technology: Politechnika Rzeszowska im Ignacego Lukasiewicza, POLAND

## Abstract

This study aims to assess the influence of internal and external factors on the Efficiency of banks in Pakistan using the Data Envelopment Analysis Approach (DEA). Bank’s Efficiency is measured through DEA Model using input and output variables. The input variable includes the number of employees, number of branches, administration expenses, non-interest expenses, and loan loss provisions. In contrast, the output variable consists of net interest income, net commissions, and total other income. This study considers the internal determinants of the bank’s Efficiency as corporate governance, enterprise risk management, ownership structure (state, foreign, and domestic ultimate owned banks), return on equity, financial leverage, and the size of the bank. The external determinants of the bank’s Efficiency include banking structure and macroeconomic conditions. The study has used data from seventeen commercial banks over the period of 2011 to 2020. The study used the Data Envelopment Analysis Approach (DEA) and Logit and Probit Regression Model to evaluate research hypotheses. The Logit model results show that corporate governance, ultimate global ownership, and return on equity have a statistically significant and positive impact on the bank’s Efficiency. Enterprise risk management and financial leverage adversely affect the bank’s Efficiency. Better corporate governance can help banks to control the risk and cost of capital and enhancement the effectiveness of capital. Similarly, better risk management of banks can lead to better operational and strategic decisions in the competitive banking environment.

## 1. Introduction

The term efficiency, particularly in the banking sector, means the best utilization of limited resources with minimum cost and maximum output. The evaluation of Efficiency helps to find how much a bank is efficient and the possible solutions to filling the gap in this respect. The banks that deliver more yields from a given amount of input are characterized as efficient banks. Therefore, applying a resource-based view of banks using input and output resources is essential to highlight a bank’s Efficiency accurately. Efficiency in financial institutions implies improved profitability, more funds channeled in, better prices, and service quality for consumers in this competitive business environment. A bank’s Efficiency may lead to bearing debt burden and benefit the well-being of depositors and routine clients. A bank’s Efficiency could play a vital role in shaping the real economy and help in inadequate economic progress. The whole economy of a country may be threatened due to a weak and incompetent banking system [1]. The efficiency measurements enable managers to benchmark the bank’s performance and explore the areas of inefficiency for future improvements [2]. The internal rating system of commercial banks using financial performance is important to support competitiveness and profitability in long term [3].

In 1975, banks in Pakistan were nationalized, and the number of banks was merged into a few larger baking institutions. In the 1990s, the government of Pakistan partially privatized NCB’s (MCB to investors and ABL to its employees & management). The government of Pakistan decided to give private participation in the banking sector and allowed the establishment of new private banks. In Pakistan, very few researchers, including [4–8] studied banking efficiency. However, most of these studies focused on the traditional Ratio Analysis Approach to measure the bank’s Efficiency. The Financial Ratios Approach has some weaknesses that necessitated adopting other methodologies. First, the Ratio Analysis Approach does not provide long-term effects. Second, many researchers argued that no one could assess the strength of a firm from a few ratios as well as the global production of the firm [5]. A recent study by [9] found that different countries face a different levels of business risk. Moreover, the Efficiency of banks brings customer satisfaction and more customers, which may translate into more profitability. However, [10] found that factors affecting bank customer satisfaction are almost the same in different countries.

[11] recently stated that there are two methods, parametric and non-parametric, to evaluate the firm’s Efficiency. The most common approach for efficiency assessment in the banking sector is the non-parametric method–Data Envelopment Analysis (DEA). The Data Envelopment Analysis model allows the Efficiency of transforming multiple inputs into multiple outputs with the help of an efficiency score. The Data Envelopment Analysis (DEA) was first introduced by [12] and it was used to calculate technical efficiencies. It has been observed that the Efficiency of the banking sector in Pakistan is not up to date in the new financial environments, especially considering the introduction of codes of corporate governance in 2012 & 2017, improved risk management framework for banks, changing regulation regarding ownership structure and other effecting factors in the modern age. The corporate governance code has introduced many new reforms, including board independence, risk management, and ownership structure. Most of the studies focused either on comparing state-owned, foreign, domestic, and Islamic banks or estimating a bank’s Efficiency through DEA and ignoring determinants, especially corporate governance and enterprise risk management. Because of these developments, there is a dire need to explore the new determinants of the banking system’s Efficiency in Pakistan using the Data Envelopment Analysis model. Banks are considered an engine of the economy, and the banks’ Efficiency may help the economy’s stability. Pakistan’s efficient financial system is an integral part of the global financial system. The research on the Efficiency of the Pakistani bank system is a good addition to finance literature because Pakistani and other developing countries’ financial system is considered inefficient.

This study uses the Data Envelopment Analysis model to explore the determinants of Pakistan’s banking industry’s Efficiency. The study includes the internal and external determinants of the Efficiency of the banking sector in the Model. The internal determinants are the Corporate Governance of the bank, Enterprise Risk Management, Ownership Structure (state, foreign, and domestic ultimate owned banks), return on equity, financial leverage, and bank size, while the external determinants of the Efficiency of banks are banking structure and macroeconomic condition in the specified Model. Recent studies evaluated bank efficinecy through DEA like [13–20] The current paper provides empirical evidence of institutional theory in the banking sector. The corporate governance practices of banks improve the Efficiency of the banks due to sound governance structure, fewer principal-agent problems, and efficient implementation of rules and regulations. This aspect of the corporate governance of banks is not much explored in literature. A bank’s Efficiency can lead to better customer service, return to depositors, fewer non-performing loans, and better economic growth and development. The Data Envelopment Analysis model has become more valuable due to Pakistan’s recent corporate governance code and risk framework introduction.

The study consists of a review of the relevant literature and the concerned theoretical framework. The following section focuses on the Model’s data, methodology, and estimation. The forthcoming section will deal with results and discussion, and in the end, the conclusion will be mentioned with some policy implications.

## 2. Theoretical background

### 2.1. The efficiency of banks: Concept and theory

The terms efficiency and productivity are used synonymously. [21] has described productivity as "the ratio between outputs and inputs." [22] defines Efficiency as "the maximum use of the existing resources in an enhanced and more productive way." In light of these definitions, it can be said that efficient firms show higher performance with minimum input. [2] defined Efficiency as "more output per unit of input indicates higher efficiency." The notion of efficiency measurement determines how a firm can maximize its output and profit while minimizing cost.

### 2.2. Types of efficiency of banks

#### 2.2.1. Cost efficiency

Cost Efficiency denotes comparing a bank’s cost to the firm’s best practices for producing the same output in the same conditions. A bank is cost-efficient if it utilizes given input at the lowest cost and produces the maximum output in a shorter period under the same conditions. According to [23], cost-efficiency refers to a minimum cost and maximum output production with limited resources. Cost Efficiency is divided into Allocative Efficiency and Technical Efficiency.

*2*.*2*.*1*.*1 Allocative efficiency*. The Allocative Efficiency denotes the use of the best level of input. According to [5] Allocative Efficiency refers to the choice of optimal input proportion at the given input prices. While according to [7] the Allocative Efficiency Change (AEC) becomes important mostly when some governance changes and state control moderates the de-regulation process. [2] claimed that Allocative Efficiency measures the optimal mix of inputs to increase Efficiency and production or services, such as introducing Automatic Teller Machines (ATM) by banks and internet banking for capital-labor tradeoffs.

*2*.*2*.*1*.*2 Technical efficiency*. Technical Efficiency refers to the maximum output production with limited time and resources. The concept of Technical Efficiency, introduced by [24] is commonly used to assess organizations. Technical Efficiency is helpful when multiple inputs and outputs are considered. Technical Efficiency is also closely related to managerial efforts. According to the production theory, Technical Efficiency is the assessment of the resources (inputs) vector used to obtain the vector of outputs. [22] claimed that Technical Efficiency indicates a good deal about the quality of managerial decisions. [2] stated that Technical Efficiency is also known as Global Efficiency. They claimed that Technical Efficiency measures the ability of banks to produce actual outputs with fewer inputs or resources used by indicating higher Efficiency. [25] focused on Technical Efficiency analysis and reported that it could indicate the quality of the management in the Russian market.

#### 2.2.2. Scale efficiency

[26] stated that production at a maximum level by utilizing the best maximum input level refers to Scale Efficiency. The overall technical efficiency ratio to pure Efficiency refers to scale efficiency [27]. In recent times, [2] have defined Scale Efficiency as "the optimal activity volume level" whereby inefficiency may arise if goods or services are produced above or below the optimal level, resulting in added fixed cost.

#### 2.2.3. Price efficiency

Efficient banks can offer better services at reasonable prices in the view of customers. At the same time, other stakeholders think that only efficient banks can ensure consistent returns. Moreover, only efficient banks can survive and maintain their market share, while in managers’ view, inefficient banks would ultimately be eliminated in changing and completive market conditions [27].

### 2.3. The efficiency of banks in Pakistan

In Pakistan, [5] believe that banks must operate efficiently in a competitive banking system. Their findings show that Government-owned banks have been observed as comparatively lower efficient than foreign banks in Pakistan from 2000 to 2005. [28] has also observed that domestic banks’ Efficiency was low compared to foreign banks. While comparing the Efficiency of foreign banks with the local banks, it has been observed that foreign banks have a higher level of Efficiency regarding ownership structure. In contrast, the Efficiency in this regard was lower in the case of domestic banks. In the opinion of [23] banking efficiency at the best level could be measured at which a bank operates by linking contribution and productivity. [8] showed that scale inefficiency governs pure technical incompetence regardless of the method used to calculate efficiency scores. Besides, it was found that 25% of incompetence in the banking sector is to their scope, which means that there is scope to progress the bank’s competence to reduce the employee numbers.

### 2.4. Determinants of the efficiency of banks in pakistan

#### 2.4.1. Corporate governance of bank and efficiency

The concept of corporate governance was first introduced by [29] and then by [30]. The concept became popular again in the recent decade, in emerging countries in the 1990s, for the better management of huge firms after the failure of certain huge firms. The relationship between corporate governance and Efficiency is significant, as better corporate governance leads to higher Efficiency in banking firms. According to [31] minimizing capital and transaction costs through better corporate governance leads to Efficiency. [32] emphasized that good corporate governance improves the firm’s technical Efficiency. [33] proposed that corporate governance principles should be adopted in the firm’s institutional environment to improve Efficiency. The board of directors is responsible for adopting and complying with corporate governance principles. [34] examined the effect of corporate governance on Efficiency and concluded that reforms in the financial sector had improved the performance of the banking industry in Pakistan. [34] has noticed that government banks are lesser efficient than private banks. Recently, [35] reported that the bank’s corporate governance is the prominent determinant of the bank’s Efficiency. [36] also suggested that bank governance boosts the bank’s Efficiency. The first hypothesis can be developed as follow;

*Hypothesis (H*_*1*_*)*: *corporate governance has a statistically significant positive impact on banks’ Efficiency*.

#### 2.4.2. Enterprise risk management and efficiency

Financial institutions have faced enormous changes, including privatization and digital banking, around the globe during the last 15 years. The possibility of unexpected incidents unfavorably disturbs the attainment of the aims or goals of an organization. Such incidents are termed "risks." Risk Management practices play a vital role in determining the behavior, Efficiency, profitability, and competitive strength of the insolvency of a banking system. [37] argued that Enterprise Risk Management (ERM) practices in a firm or financial institution are a critical concept after the financial crisis of 2007–2008. Enterprise Risk Management becomes the hot and far-front interest of the top management, government regulators, and other stakeholders. The reasons include the increase in the default rate and awareness of risk, which results in better operational and strategic decisions.

Enterprise Risk Management helps firms and other institutions minimize the risk and cost of capital, improve the Efficiency of capital, and create synergies between various risk management techniques. David Walker has strongly recommended better governance and risk management practices for an efficient banking system. In Pakistan, [38] found that higher risk of banks increases the profit efficiency and decreases the cost efficiency due to more contribution in generating revenue than inflating costs. However, in China, [39] found that a moderate level of risk preference is the appropriate approach to attain Efficiency. The second hypothesis can be developed as follow;

*Hypothesis (H*_*2*_*)*: *Higher risk management practices lead to an efficient banking system*.

#### 2.4.3. Ownership structure and efficiency

The corporate governance code and other financial sector reforms have changed the banking sector’s ownership structure during the last two decades. State-owned banks used to be dominated previously in the banking sector but currently, the ownership structure is more concerned with the shareholder percentage of control in the banking sector [40]. To compare different types of bank ownership, [41] have suggested that foreign banks have better Efficiency than other banks. [42] findings align with the theoretical argument claiming that foreign ownership brings better governance and monitoring practices, which is consistent with agency theory. Efficiency and performance were reduced in all types of banks during the financial crisis in emerging countries [43]. In China, [39] found that state-owned banks were less efficient during the financial crisis. The Third hypothesis can be developed as follow;

*Hypothesis (H*_*3*_*)*: *State ownership has an adverse but significant impact on the bank’s Efficiency*.

#### 2.4.4. Bank size and efficiency

The size of a bank is an essential element that can be measured through the total assets of the bank. [44] have shown a positive correlation between the size and Efficiency of banks. [45] in their study, have concluded that economies of scale are attained easily because large size reduces cost and ability to gather information for the running of the business. The log of total assets in the measurement is used by [46] in Pakistan and has shown a significant impact on the size of a bank. The fourth hypothesis can be developed as follow;

*Hypothesis (H*_*4*_*)*: *Bank size positively impacts bank efficiency*.

#### 2.4.5. Return on Equity (ROE) and efficiency

Return on equity refers to the return on the volume invested by the shareholder and their income and profit. It is also assuming the response to the shareholder’s equity. If the ratio is higher, it means higher income generation and better profit for the company [47]. The result has shown that the Efficiency and performance of the banks were reduced in both emerging and developed countries during the global financial crisis. [48] have observed a negative relationship between Efficiency and Return on Equity in Tunisia. The fifth hypothesis can be developed as follow;

*Hypothesis (H*_*5*_*)*: *Return on equity positively impacts banks’ Efficiency*.

#### 2.4.6. Financial leverage (total assets to total equity) and efficiency

Banks’ assets are used as collateral because of lower agency costs, which may be linked to using debt. Therefore, the financial leverage proxy is measured from the ratio of total assets to total equity (TATE). The ratio indicates the relationship of the total assets with the shareholders-owned proportion with the financial leverage/debt extensive to bank finance. In the short term, assets are preferred over debt if a firm is in the growth stage. However, in the long run, it is beneficial to increase the funds of a bank because of the relationship between financial leverage (TATE) and the bank’s performance in terms of Efficiency, which is positive. The sixth hypothesis can be developed as follow;

*Hypothesis (H*_*6*_*)*: *Financial leverage has a positive impact on a bank’s Efficiency*.

#### 2.4.7. Macroeconomic conditions and efficiency

External factors are not under the management’s control; consequently, macroeconomic conditions are mainly in refining efficiency. The external factors that can cause devastating effects, no matter how healthy the controls of the banks are, refer to the macroeconomic conditions. These are alleged to be fatal for the financial crises that have occurred previously due to overpowering. [49] has claimed that the impact of macroeconomic conditions exists at the short and long-term levels in developing countries. The macroeconomic factors, i.e., inflation, interest rate, etc., significantly affect the ownership and financial recital [46]. The seventh hypothesis can be developed as follow;

*Hypothesis (H*_*7*_*)*: *Macroeconomic Conditions have an impact on a bank’s Efficiency*.

#### 2.4.8. Banking structure of the financial system and efficiency

Generally, in most industries, competition is considered a positive force for the Efficiency and development of an organization. Competition in banks leads to Efficiency in operations and financial system soundness [50]. [51] have observed that the entry of foreign banks into the domestic market can increase the competition. Domestic banks may start competing with foreign banks if the government-owned banks are ineffective in their operations. Consequently, increasing the number of banks in a market increases the sense of competition and concentration and improves Efficiency because it can encourage banks to become more efficient. [52] have claimed that adverse selection could be through a competitive environment, which results in a decrease in profitability and Efficiency in the system. The eighth hypothesis can be developed as follow;

*Hypothesis (H*_*8*_*)*: *banking structure has an impact on a bank’s Efficiency*.

### 2.5. Theoretical framework

**Fig 1:**
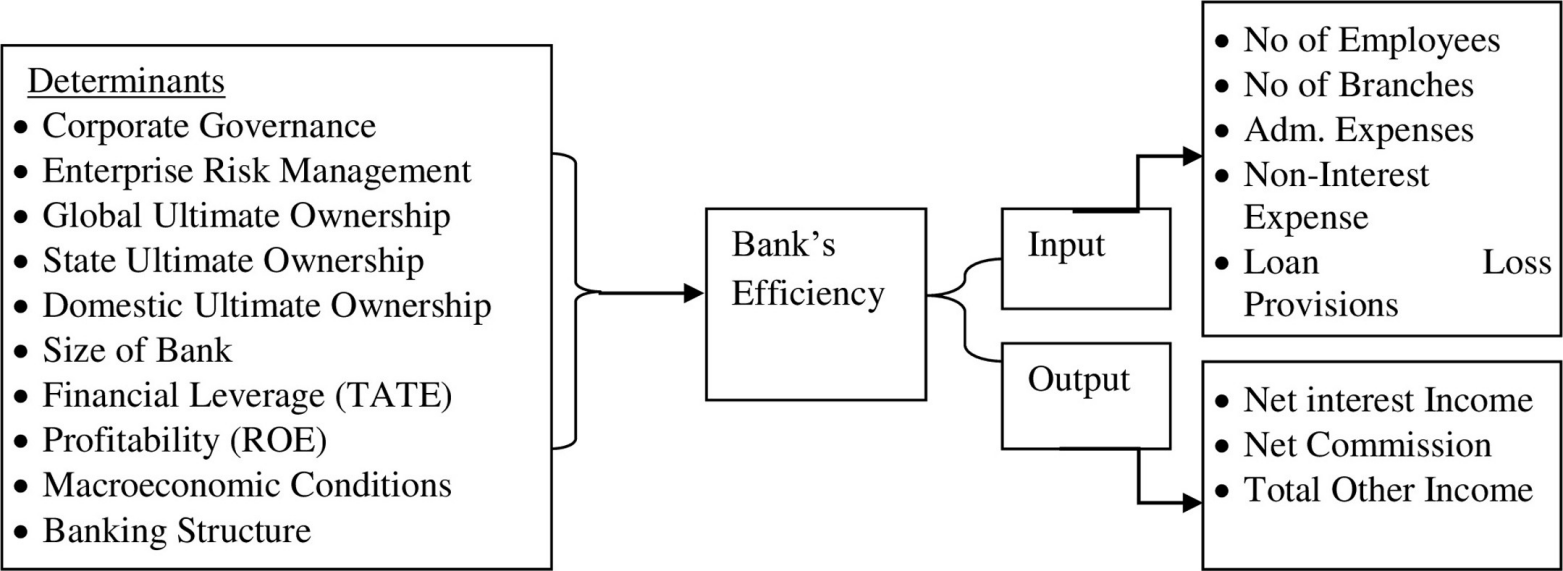
Theoretical framework (Source: Author’s demonstration).

The current study conceptual model framework of independent determinants and dependent variable banks Efficiency based on input and output variables demonstrated in Fig 1 below.

## 3. Research methodology, data measurement, and model

### 3.1. Data and measurement

The total population of listed commercial banks in Pakistan is 20 as of December 31, 2021(PSX, 2021). The longitudinal data were gathered from the annual report of 17 commercial banks in Pakistan for 2011–2020. The three banks were deleted due to incomplete data. The bank’s efficiency-dependent variable has a binary score of one and zero generated through the DEA approach. The input variable includes the number of employees, branches, administration expenses, non-interest expenses, and loan loss provisions. All values are normalized and converted into zero and one. The output variable consists of net interest income, commissions, and other income.

The corporate governance score is calculated considering the composition of committees as board size, board independence, Chief Executive Officer/ Chairman duality, managerial ownership for internal control, and audit committee independence characteristics which are measured through the dummy variables by giving the value of 1 and 0. The total number of directors demonstrates the size of the board. The board size effect is captured using a dummy variable (1 if the sample’s median is greater than 60% or 0 otherwise). Each bank’s independent director’s percentage is used as a proxy to measure the board’s Independence. The effect of the board independence is calculated through a dummy variable (1 if the independent director’s percentage is greater than 75%, 0 otherwise).

The third characteristic of corporate governance refers to the proxy of the Chief Executive Officer/ Chairman duality when the CEO is also the board chairperson, if not any lead director in each bank. The impact of the chairman duality is captured by using a dummy variable (1 in the case when the chairman and CEO are the same people, 0 otherwise). The fourth characteristic is managerial ownership, which also shows internal control. Managerial ownership is calculated as the percentage of shares held by executive directors and management at the senior level divided by each bank’s total shares. In the second step, the dummy variable (0 if the sample size percentage is higher than the sample’s median, one otherwise) is used to measure the impact of managerial ownership. The fifth characteristic is audit committee independence refers to each independent bank director’s numbers. The effect of the Independence of the audit committee is captured by a dummy variable (1 if independent directors, including the chairman of the audit committee, are greater than 75%, 0 otherwise).

Standard and Poor’s credit rating score of each bank has been used as the proxy of enterprise risk management, as suggested by [37, 53]. The log of total assets is used as a proxy to measure the bank size. The effects of the types of ownership on the bank’s Efficiency are calculated using four different dummy variables. I. 1 if the banks are State-Owned Banks, 0 otherwise. II. If the banks are the Domestic Ultimate Owned banks, 0 otherwise. III. If the banks are private/foreign banks, 0 otherwise). VI. 1 if the banks are global-owned, 0 otherwise. Data on the type of ownership structure was gathered from the bank focus (formerly bank scope).

ROE refers to the proportion of net income after payment of taxes divided by total equity, and data is collected from the annual financial statement. Financial leverage was calculated through the proxy of total assets to total equity. Credit growth is used as a proxy to measure the domestic credit to the private sector (% of GDP). The real interest rate is used as a proxy to measure the macroeconomic conditions, which were measured through a seasonal change of real interest rate (see [54]). The data is gathered from the database of the World Bank, WDI. The proxy of bank concentration and competition refers to the banking structure by adopting the study of [54]. Concentration is measured by considering the assets of the three largest banks divided by the banking sector’s total assets. The data has been gathered from the World Bank, Financial Structure, and Development database. The banking structure is measured by using a proxy of the foreign bank competition as a global share of the banking sector assets and the degree of foreign bank entry. The data has been gathered from the economic freedom of the World Database.

### 3.2. Estimation of the model

[26] has presented the latest frontier approach for judging inefficiency by stating, "the divergence of actual real from optimal most favorable behavior." In the Frontier approach further, four types: SFA stands for Stochastic Frontier Analysis, TFA stands for Thick Frontier Approach, and DFA stands for Distribution-Free Approach are parametric methodologies. Fourth, DEA stands for Data Envelopment Analysis, which is a non-parametric methodology. According to a recent study by [55] there are parametric and non-parametric methods for efficiency assessment. The most common approach in the banking sector is the non-parametric method, like the DEA model, to measure the bank’s Efficiency. The DEA approach was first introduced by [12] and they used it for calculating technical efficiencies. Many parametric methodologies require a larger sample size, and the DEA method also works well for a small sample size.

In the empirical literature, [56] used a DEA to estimate the performance efficiency of U.S. credit unions in 2009. They have stated that in recent years, DEA has become one of the popular measurement methodologies for assessing Efficiency in financial institutions. They have suggested that DEA identifies the most proficient input-output combinations. Similarly, it develops the best practice efficiency frontier against peers. The DEA technique evaluates the performance using Tobit, and Probit, to investigate whether Federal Credit Unions (FCUs) and Federally Insured State-Chartered Credit Unions (FISCUs) react differently to market-wide economic shocks. Suppose the Efficiency of banks is looked into without using the DEA approach. In that case, it is hard to suggest recommendations because, in this approach, the capability to treat with several inputs and output is consistent. The banks that performed better consider efficient, and vice versa. Data Envelopment Analysis (DEA) in Pakistan is not frequently used to investigate banks’ Efficiency because most studies focus on financial profitability. In addition, most studies focused on either comparing state-owned, foreign, domestic, and Islamic banks or just estimating a bank’s Efficiency through DEA and ignoring determinants, especially corporate governance and enterprise risk management.

In this study, we have tried to explore bank efficiency determinants by considering bank-specific and external factors. The study used the DEA score of bank efficiency as the estimated Model’s dependent variable. The paper is a fresh perspective on the bank’s Efficiency in Pakistan through the DEA approach and considering important determinants like corporate governance and risk management. DEA is generally used to calculate the production limit. DEA models are among the quantitative models that express the analyzed entities’ relative efficiency scores. The Model fully embodies the definition of Efficiency [57]. In DEA models, the indicators are divided into two groups, the inputs and outputs. The input satisfies the minimization criterion, and the outputs fulfill the maximization criterion. [25] have stated that Data Envelopment Analysis looks at individual companies (in our case, banks) and their relative performance compared with their competitors. Therefore, for banks, technical efficiency scores can be used to monitor the dynamics and potential of each bank.


Bank'sEfficiency=DEAScore=Output/Input

### 3.3. Logit and probit regression model

Using the DEA approach, homogeneity among banks was expected in assessing a bank’s effectiveness. The present study used a discrete dependent variable model, the "Logit Regression Model," with a binary type variable (Logit Module, 2005). The Logit regression model has been used as a second step with the DEA approach [58]. This Model is used. The dependent variable is binary or dichotomous, taking two values, 0 and 1 showing the likelihood of occurrence of an event. In the Logit model, the likelihood of an event is occurred expressed as;

$$P_{i}=E\left(Y_{i}=\frac{1}{X_{i}}\right)=\frac{1}{1+e^{-\left(\beta 1+\beta 2Xi\right)}}$$ (1)

This equation can also be described as follows;

$$P_{i}=\frac{1}{1+e^{-Zi}}=\frac{e^{Z}}{1+e^{-Zi}}$$

Where,

$$Z_{i}=\beta_{i}+\beta_{i}X_{i}$$ (2)

Let Pi is the probability that an event has occurred, and (1 -Pi) is the probability that not an event occurs. Now consider the following Model of (1 -Pi):

$$1-P_{i}=\frac{1}{1+e^{Zi}}$$

So,

$$\frac{P_{i}}{1-P_{i}}=\frac{1+e^{Zi}}{1+e^{-Zi}}=e^{Zi}$$

This natural ratio log (Li) is called the logit. Therefore, the Model is referred to as the Logit model.

Now consider the following Model:

$$L_{i}=\ln\left(\frac{P_{i}}{1-P_{i}}\right)=Z_{i}=\beta_{i}+\beta_{i}X_{i}$$ (3)

The Logit Model revealed that the log of the odds ratio is a linear function of explanatory variables. In this Model, the slope coefficient βi gives the change in the log of the odds ratio per unit change in the Xi. Furthermore, for the validation of the estimated results, this study has also used the Probit Model, which is applied where the dependent variable can take only two variables, such as input and output. In the probit model probability, the inverse standard normal distribution is modeled as a linear combination of the predictors.

The dependent variable is the bank’s Efficiency, and this is the score given by DEA. The value of these scores is between one and less than one but greater than zero. Therefore we have transformed all the values ≥ one as one otherwise zero. Moreover, we have determined the impact on banks’ Efficiency of some firm-specific and external variables, including corporate governance, enterprise risk management, state-owned ultimate banks, domestic-owned ultimate banks, global-owned ultimate banks, banks size, return on equity, financial leverage (TATE), size of the banks, macroeconomic conditions and banking. By including the explanatory variables in the equation of Efficiency Logit Model 3 and econometrically, the models are expressed as follows;

$$\begin{array}{l} {\text{EfficiencyLogit}}_{\text{i},\text{t}}\\ =\beta_{0+}\sum_{n=1}^{\infty }\beta_{1}\left(CG_{i,t}\right)+\sum_{n=1}^{\infty }\beta_{2}\left(ERM_{i,t}\right)+\sum_{n=1}^{\infty }\beta_{3}\left(SB_{i,t}\right)+\sum_{n=1}^{\infty }\beta_{4}\left(GUO_{i,t}\right)\\ +\sum_{n=1}^{\infty }\beta_{5}\left(SUO_{i,t}\right)+\sum_{n=1}^{\infty }\beta_{6}\left(DUO_{i,t}\right)+\sum_{n=1}^{\infty }\beta_{7}\left(TATE_{i,t}\right)+\sum_{n=1}^{\infty }\beta_{8}\left(ROE_{i,t}\right)\\ +\sum_{n=1}^{\infty }\beta_{9}\left(MC_{i,t}\right)+\sum_{n=1}^{\infty }\beta_{10}\left(BS_{i,t}\right)+\mu_{i,t} \end{array}$$ (4)

$$\begin{array}{l} {\text{EfficiencyProbit}}_{\text{i},\text{t}}\\ =\beta_{0+}\sum_{n=1}^{\infty }\beta_{1}\left(CG_{i,t}\right)+\sum_{n=1}^{\infty }\beta_{2}\left(ERM_{i,t}\right)+\sum_{n=1}^{\infty }\beta_{3}\left(SB_{i,t}\right)+\sum_{n=1}^{\infty }\beta_{4}\left(GUO_{i,t}\right)\\ +\sum_{n=1}^{\infty }\beta_{5}\left(SUO_{i,t}\right)+\sum_{n=1}^{\infty }\beta_{6}\left(DUO_{i,t}\right)+\sum_{n=1}^{\infty }\beta_{7}\left(TATE_{i,t}\right)+\sum_{n=1}^{\infty }\beta_{8}\left(ROE_{i,t}\right)\\ +\sum_{n=1}^{\infty }\beta_{9}\left(MC_{i,t}\right)+\sum_{n=1}^{\infty }\beta_{10}\left(BS_{i,t}\right)+\mu_{i,t} \end{array}$$ (5)

Where Efficiency is banking outputs and inputs, C.G. represents the corporate governance composite, ERM indicates enterprise risk management, S.B. represents the size of the bank, GOU indicates the global ultimate owned banks, SUO indicates state-owned ultimate banks, DOU indicates domestic-owned ultimate banks, TATE represents the financial leverage of the banks, ROE indicates the return on equity, MC represents the macroeconomics conditions. B.S. demonstrates the banking structure of the banks. *β*_0_ indicate the constant unknown parameters to be estimated while *μ* is the error term where "t" denotes years as time and a particular of banks **"**i" i = 1,2,…, N.

## 4. Results and discussion

### 4.1. Descriptive summary

Table 1 shows descriptive statistics that demonstrate the complete comprehension of the sample variables, such as Efficiency through input and output and its relationship with other important factors like corporate governance, enterprise risk management, ownership structure, size of the bank, financial leverage, return on equity, macroeconomic conditions, and banking structure from 2011 to 2020. Descriptive based on means indicates the central tendency of all variables based on 168 observations. The bank’s Efficiency (i.e., dependent variable) mean value is 0.83. Moreover, all other independent variables’ mean value ranges from 0.04 to 18.69. The Jarque-Bera test indicates the normal distribution of data. All variable probability values demonstrate that values are significant with p-value less than 1 and 5% except for the size of banks. The detailed results are shown in Table 1.

**Table 1:** Results of descriptive statistics.

Variables	Mean	Jarque-Bera	Probability	Observations
Bank’s Efficiency	0.83	29404.48	0.00	168.00
Corporate Governance	3.26	14.85	0.00	168.00
Enterprise Risk Management	4.27	11.67	0.00	168.00
Size of Bank	12.70	4.49	0.11	168.00
Global Ultimate Ownership	0.53	28.00	0.00	168.00
State Ultimate Ownership	0.18	83.56	0.00	168.00
Domestic Ultimate Ownership	0.59	28.12	0.00	168.00
Financial Leverage (TATE)	0.04	4794.46	0.00	168.00
Profitability (ROE)	6.40	4620.20	0.00	168.00
Macroeconomic Conditions	18.69	67.31	0.00	168.00
Banking Structure	5.24	9.94	0.01	168.00

Source: Author’s Estimation

### 4.2. Data envelopment analysis graph

**Fig 2:**
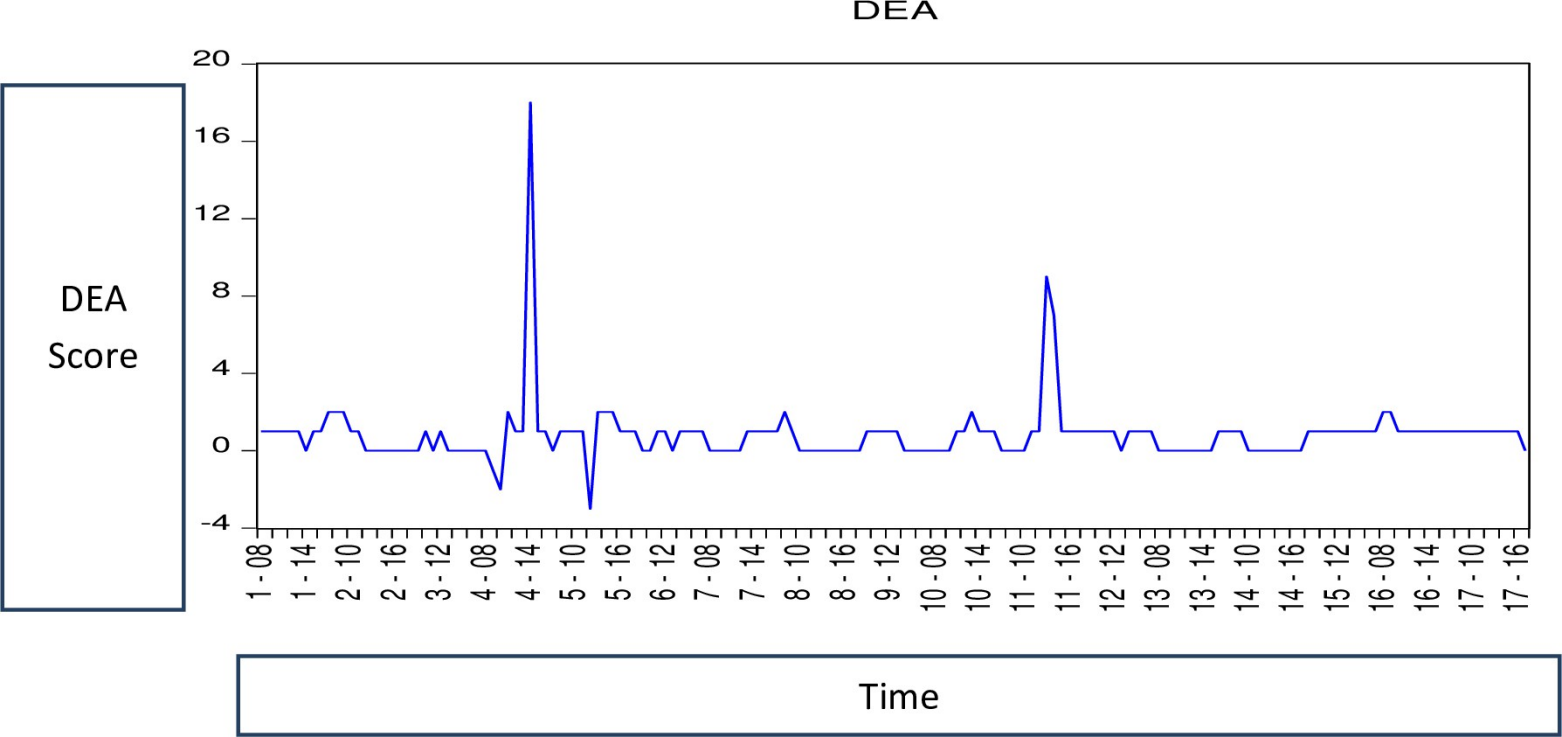
Representation of DEA results (Source: Author’s estimations).

The orientation system can be designated as input orientation or output orientation. An input orientation means how much inputs can be reduced to get current optimal level outputs for a firm that can help it be DEA-efficient. While an output orientation helps to find how much we can increase the output through consuming a current level of inputs so that a firm can be among the DEA-efficient ones. The representation of DEA results is shown in Fig 2.

### 4.3. Results of determinants of bank’s efficiency through logit and probit models

The estimated results, based on Logit and Probit models, are summarized in Table 2. The results in Table 2 showed that the McFadden R- squared value is positive and significant for both models. McFadden’s R2 can be defined as 1−LLmod/LL0 because a fitted model log of likelihood values is LLmod while LL0 is for the null model likelihood log of likelihood. This includes only an intercept as a predictor; therefore, every individual has predicted the same probability of ’success.’ The McFadden R-squared value of the Logit model is 0.15, and the Probit Model value is the same as the Logit model. The cost of the L.R. statistic is high and positive for both models, such as the L.R. statistic is 30.89 for the Logit model and the Probit model with a minor value change of 30.94.

**Table 2:** Results of efficiency logit and probit model.

Variable	Logit Model			Probit Model		
	Coefficient	z-Statistic	Prob.	Coefficient	z-Statistic	Prob.
Constant	22.57	3.36	0.00	13.63	3.46	0.00
Corporate Governance	0.47	1.67	0.09	0.28	1.69	0.09
Enterprise Risk Management	-1.84	-3.16	0.00	-1.13	-3.26	0.00
Size of Bank	-0.83	-3.26	0.00	-0.50	-3.36	0.00
Global Ultimate Ownership	0.77	1.62	0.10	0.48	1.69	0.09
State Ultimate Ownership	-1.84	-2.50	0.01	-1.12	-2.57	0.01
Domestic Ultimate Ownership	-0.47	-0.80	0.42	-0.26	-0.76	0.45
Financial Leverage (TATE)	-2.13	-1.63	0.10	-1.19	-1.57	0.12
Profitability (ROE)	0.00	0.41	0.68	0.00	0.40	0.69
Macroeconomic Conditions	-0.07	-1.23	0.22	-0.05	-1.29	0.20
Banking Structure	-0.67	-1.21	0.23	-0.40	-1.20	0.23
LR statistic	30.89		0.00	30.94		0.00
McFadden R-squared	0.15			0.15		

Source: Author’s Estimation; Dependent Variable: Banks’ Efficiency

The Logit and robust probit model results show that the Model constant coefficient value, i.e., 22.57 and 13.63, respectively, is significant and positive with a p-value less than 1%, which indicates that the banking efficiency is based on output and input in Pakistan. Overall, the coefficient, Z-statistics and probability values of the Model indicate that Pakistani banks are on an efficiency track. The Logit model results in Table 2 show that corporate governance has a positive and statistically significant effect on the bank’s Efficiency with a p-value less than 10%. Therefore, findings endorse the alternate hypothesis (H_1_) that corporate governance has a statistically significant positive impact on banks’ Efficiency. Enterprise risk management and financial leverage showed adverse effects on Efficiency, whereas enterprise risk management showed a negative but significant p-value of less than 1%.

In contrast, financial leverage showed a negative but insignificant impact on Efficiency, which indicates that a higher level of leverage and lacking risk management process leads to inefficiency. Therefore, the findings of hypothesis (H_2_) failed to reject the null hypothesis in favor of higher risk management practices leading to an efficient banking system. Also, hypothesis (H_6_) based on financial leverage failed to reject the null hypothesis means that in Pakistan, higher risk-taking practices hurt the banking efficiency and profitability of return on equity.

In addition, the findings indicate that ultimate global ownership positively influences the banks’ Efficiency. Therefore, findings endorse and accept the alternate hypothesis (H_3_) for the globally owned banks. However, State ownership has an adverse but significant impact on the bank’s Efficiency, with a p-value of less than 5%. Moreover, domestically owned banks have an adverse and insignificant impact on banks’ Efficiency. It means that in Pakistan, globally-owned banks are more efficient than state-owned and domestic banks. The size of the bank also has a negative impact on its Efficiency of the bank. The findings of hypothesis (H_4_) accept the null hypothesis by rejecting the alternate that bank size positively impacts bank efficiency.

Moreover, return on equity (ROE) show a significant and positive but partial impact on banks’ Efficiency, therefore, endorsing the alternate hypothesis (H_5_). Findings indicate that larger banks are inefficient in Pakistan as the size of the banks indicates a negative but significant return on particular equity impact on banks’ Efficiency, this lead toward smaller banks’ Efficiency. The macroeconomic conditions and banking structure show a negative and insignificant impact on banking efficiency. Therefore, findings of hypotheses (H_7_) and (H_8_) endorse the result’s consistency with the literature as macroeconomic conditions such as real interest rate, inflation, and economic freedom are inherent in the financial and banking system and hurt the Efficiency of the banks. The study results are robust as the Probit Model also produced the same results with minor values changes, indicating the validity and reliability of the Model fit for use.

### 4.4. Discussion of results

The fact that corporate governance improvement leads to a bank’s Efficiency is consistent with the literature. As per the institutional theory, corporate governance improves Efficiency through decision-making. Therefore, this paper empirically proved the application of institutional theory in the banking industry. The banking sector in Pakistan has recently faced tough competition and introduced much new technology, including online banking. This fact makes Efficiency in the banking sector an important policy agenda. The introduction of the corporate governance code in 2012 & 2017 was also a policy initiated in the wake of these problems. The results of the current study are chiefly consistent with the empirical findings of [32, 33]. In addition, this study found that foreign banks are more efficient than state-owned in Pakistan, thus confirming the earlier findings [5, 34] Similarly, banks with ultimate global owners are more efficient than domestic banks in Pakistan, confirming the findings [28].

The results showed that poor risk management practices result in inefficiency in the banking sector. The results are consistent with [38]. They found that a higher risk of banks decreases the cost efficiency due to more contribution in generating revenue than inflating costs. Risk management always suggests taking a reasonable risk for efficient operations and performance. The higher risk may endanger the bank’s Efficiency due to higher costs associated with higher risk. This result supported the findings of [39] who found that a moderate level of risk preference is the appropriate approach to attain Efficiency.

Furthermore, the study results show a negative relationship between enterprise risk management (better risk management) and Efficiency (calculated output/input). Better risk management always results in incremental costs for banks. Hence, a negative sign appears in the coefficient of enterprise risk management which indicates that in Pakistan, banks are not taking a moderate risk, and stakeholder force managers to take higher risks to maximize the profit. The size of the bank also has a positive and statistically significant impact on its Efficiency. The results contradict the findings of [45, 46]. They proposed that economies of scale result in a positive relationship between the size and Efficiency of the bank. However, the changing environment, innovative technologies, and online banking change the relationship between the size and Efficiency of the banks. The results confirm that lean and smart banks are more efficient than large and traditional banks. These results are a fresh perspective on banks’ Efficiency in changing and competitive business environments, like the introduction of a new code of corporate governance, reforms in risk management and ownership structures, and lean structure of banks.

## 5. Conclusion and policy implications

The study aims to determine the effects of corporate governance, risk management, type of ownership, macroeconomic conditions, and banking structure on the bank’s Efficiency in the Pakistan banking sector from 2011–2020 based on panel data. The study used the Data Envelopment Analysis (DEA) approach to measure the Efficiency of the banks based on output and input variables. The study applied the Logit and Probit models to explore the effects of various determinants on the Efficiency of banks. The estimated results showed that improved corporate governance led to Efficiency in the bank sector of Pakistan, which is consistent with the extant literature. The DEA model suggests that banks that deliver a high yield from a given amount of information are characterized as efficient banks. Therefore, applying a resource-based view of banks using input and output resources is essential to highlight the accurate picture of Efficiency.

The findings suggest that efficiency measurement enables managers to benchmark a bank’s overall performance. Better corporate governance of banks enables bank management to make better and cost-effective decisions and improve Efficiency in terms of the ratio of output and input. Better risk management may help banks and institutes control risk, reduce the cost of capital, enhance the effectiveness of capital, and create synergies between various enterprise risk management techniques. Better risk management of managers can lead to better operational and strategic decisions. For a rigorous and competitive banking sector in the country, it is essential to attain macroeconomic goals. The external factors can cause devastating effects, no matter how healthy the controls of the banks are, which refer to the macroeconomic conditions alleged for the financial crises. The findings demonstrate that a slowdown in macroeconomic growth and insufficient demands at a domestic and international level have caused the decline of Efficiency in banks.

The paper provides empirical evidence of institutional theory in the banking sector. The corporate governance practices of banks improve the Efficiency of the banks due to sound governance structure, fewer principal-agent problems, and efficient implementation of rules and regulations. This aspect of the corporate governance of banks is not much explored in literature. The regulator should ensure strict compliance with the code of corporate governance risk management side of the banks and encourage global ownership in the banking sector in Pakistan. The regulator should encourage competition as it boosts the services which are value-added for the satisfaction of customers as well as profitable for the bank. Sound risk practices during competition are critical for an efficient banking system. A bank’s Efficiency can lead to better customer service, return to depositors, fewer non-performing loans, and better economic growth and development. Future research may be untapped on the role of financial technology in banking efficiency. Moreover, future work may compare the data envelopment analysis with other measures. Also, future studies can explore the banking efficiency with the banking competition concerning regional countries’ banking systems.

## Supporting information

S1 File DAE paper dataset. (XLSX)

S2 File List of bank. (DOCX)
