# Supplementary material for: Determinants of bank’s efficiency in an emerging economy: A data envelopment analysis approach
Source: PLoS One. 2023 Mar 14;18(3):e0281663. doi: 10.1371/journal.pone.0281663 (PMC10027419; doi:10.1371/journal.pone.0281663)
Supplement: S2 File — (DOCX) [file pone.0281663.s002.docx]

| List of Banks | |
| --- | --- |
| 1 | Allied Bank Limited |
| 2 | Albaraka Bank Limited |
| 3 | Bank Al-Habib |
| 4 | Bank Alfalah Limited |
| 5 | Bank Islami |
| 6 | Bank of Khyber |
| 7 | Bank of Punjab |
| 8 | Faysal Bank Limited |
| 9 | Habib Bank Limited |
| 10 | JS bank |
| 11 | MCB |
| 12 | Meezan Bank |
| 13 | NBP |
| 14 | Silk Bank Limited (Saudi Pak Commercial Bank Limited) 07 |
| 15 | Soneri Bank Limited |
| 16 | Summit Bank |
| 17 | UBL |
